# (*E*,*E*)-*N* ^1^,*N* ^2^-Bis(2,6-difluoro­benzyl­idene)ethane-1,2-diamine

**DOI:** 10.1107/S1600536811044692

**Published:** 2011-10-29

**Authors:** Mohammad Khaledi Sardashti, Reza Kia, William Clegg, Ross W. Harrington

**Affiliations:** a Department of Chemistry, Faculty of Science, Islamic Azad University, Shahrekord Branch, Box 166, Tehran, Iran; b Department of Chemistry, Science and Research Branch, Islamic Azad University, Tehran, Iran; c School of Chemistry, Newcastle University, Newcastle upon Tyne NE1 7RU, England

## Abstract

The asymmetric unit of the title compound, C_16_H_12_F_4_N_2_, comprises half of the potentially bidentate Schiff base ligand, with an inversion centre located at the mid-point of the central C—C bond. The crystal packing is stabilized by inter­molecular C—H⋯N and π–π inter­actions [centroid–centroid distance = 3.6793 (12) Å and inter­planar spacing = 3.4999 (7) Å].

## Related literature

For background to the synthesis and structural variations of Schiff base ligands and their complexes, see: Granovski *et al.* (1993 ▶); Elmali *et al.* (2000 ▶).
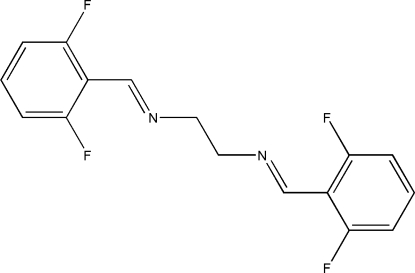


## Experimental

### 

#### Crystal data

C_16_H_12_F_4_N_2_
*M* *_r_* = 308.28
Monoclinic, 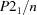

*a* = 7.3304 (10) Å
*b* = 10.5414 (15) Å
*c* = 9.2106 (13) Å
β = 105.487 (2)°
*V* = 685.89 (17) Å^3^
*Z* = 2
Mo *K*α radiation
μ = 0.13 mm^−1^
*T* = 150 K
0.34 × 0.30 × 0.10 mm

#### Data collection

Bruker SMART 1K CCD area-detector diffractometer
Absorption correction: multi-scan (*SADABS*; Bruker, 2005 ▶) *T*_min_ = 0.958, *T*_max_ = 0.987
4573 measured reflections
1203 independent reflections
1057 reflections with *I* > 2σ(*I*)
*R*_int_ = 0.031

#### Refinement

*R*[*F* ^2^ > 2σ(*F* ^2^)] = 0.043
*wR*(*F* ^2^) = 0.124
*S* = 1.15
1203 reflections
100 parameters
H-atom parameters constrained
Δρ_max_ = 0.27 e Å^−3^
Δρ_min_ = −0.23 e Å^−3^

### 

Data collection: *SMART* (Bruker, 2005 ▶); cell refinement: *SAINT* (Bruker, 2005 ▶); data reduction: *SAINT*; program(s) used to solve structure: *SHELXS97* (Sheldrick, 2008 ▶); program(s) used to refine structure: *SHELXL97* (Sheldrick, 2008 ▶); molecular graphics: *SHELXTL* (Sheldrick, 2008 ▶); software used to prepare material for publication: *SHELXTL* and *PLATON* (Spek, 2009 ▶).

## Supplementary Material

Crystal structure: contains datablock(s) global, I. DOI: 10.1107/S1600536811044692/su2335sup1.cif

Structure factors: contains datablock(s) I. DOI: 10.1107/S1600536811044692/su2335Isup2.hkl

Supplementary material file. DOI: 10.1107/S1600536811044692/su2335Isup3.cml

Additional supplementary materials:  crystallographic information; 3D view; checkCIF report

## Figures and Tables

**Table 1:** Hydrogen-bond geometry (Å, °)

*D*—H⋯*A*	*D*—H	H⋯*A*	*D*⋯*A*	*D*—H⋯*A*
C5—H5⋯N1^i^	0.95	2.53	3.471 (2)	171

Symmetry code: (i) 
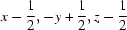
.

**Fig. 1.**
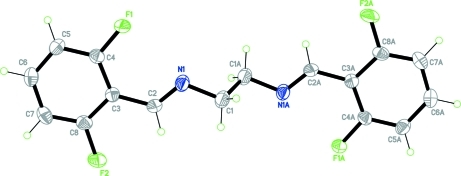
The molecular structure of the title compound, showing 50% probability displacement ellipsoids and the atomic numbering. Symmetry code for suffix A: -x + 1, -y + 1, -z + 2.

**Fig. 2.**
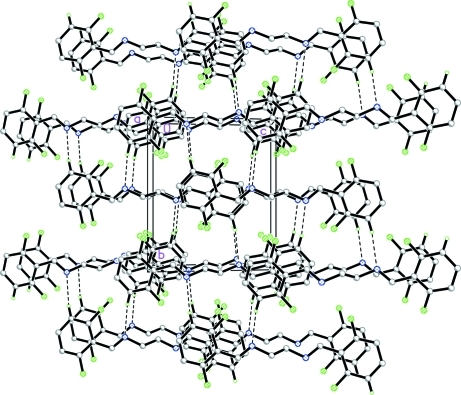
The crystal packing, viewed down the a axis, showing linking of molecules through the intermolecular C—H···N hydrogen bonds to form sheets in the (1 0 - 1) plane. The dashed lines represent these intermolecular interactions.

## supplementary crystallographic information

### Comment

Schiff base ligands are among the most prevalent ligands in the field of coordination chemistry. Metal derivatives of Schiff bases have been studied extensively, and Ni^II^ and Cu^II^ complexes play a major role in both synthetic and structural research (Elmali *et al.*, 2000; Granovski *et al.*, 1993).

The asymmetric unit of the title compound comprises half of the potentially bidentate Schiff base ligand; an inversion centre is located in the middle of the central C—C bond (Fig. 1). Each half of the molecule is essentially planar, the imine segment (C1—N1═C2—C3) being rotated only 7.36 (10)° out of the plane of the benzene ring. The two halves are parallel by inversion symmetry, but not coplanar, the CH_2_CH_2_ linker unit forming a step between them.

The crystal packing is stabilized by intermolecular C—H···N interactions (see Table 1), which generate sheets parallel to (1 0 -1), and by intermolecular π-π interactions [centroid-centroid distance = 3.6793 (12) Å, interplanar separation = 3.4999 (7) Å, the two planes are strictly parallel by inversion symmetry].

### Experimental

The title compound was synthesized by mixing 2,4-difluorobenzaldehyde (4 mmol) and ethylenediamine (2 mmol) in chloroform (20 ml). After stirring for 2 h, the solution was filtered and the resulting yellow solid was crystallized from ethanol, giving single crystals suitable for X-ray diffraction.

### Refinement

All H atoms were positioned geometrically and constrained to ride on the parent atoms, with C—H = 0.95–0.99Å and *U*_iso_(H) = 1.2 *U*_eq_(C).

### Crystal data

**Table:**

C_16_H_12_F_4_N_2_	*F*(000) = 316
*M**_r_* = 308.28	*D*_x_ = 1.493 Mg m^−^^3^
Monoclinic, *P*2_1_/*n*	Mo *K*α radiation, λ = 0.71073 Å
Hall symbol: -P 2yn	Cell parameters from 4055 reflections
*a* = 7.3304 (10) Å	θ = 2.9–28.3°
*b* = 10.5414 (15) Å	µ = 0.13 mm^−^^1^
*c* = 9.2106 (13) Å	*T* = 150 K
β = 105.487 (2)°	Plate, yellow
*V* = 685.89 (17) Å^3^	0.34 × 0.30 × 0.10 mm
*Z* = 2	

### Data collection

**Table:**

Bruker SMART 1K CCD area-detector diffractometer	1203 independent reflections
Radiation source: fine-focus sealed tube	1057 reflections with *I* > 2σ(*I*)
graphite	*R*_int_ = 0.031
Detector resolution: 8.33 pixels mm^-1^	θ_max_ = 25.0°, θ_min_ = 3.0°
ω scans	*h* = −8→8
Absorption correction: multi-scan (*SADABS*; Bruker, 2005)	*k* = −12→12
*T*_min_ = 0.958, *T*_max_ = 0.987	*l* = −10→10
4573 measured reflections	

### Refinement

**Table:**

Refinement on *F*^2^	Primary atom site location: structure-invariant direct methods
Least-squares matrix: full	Secondary atom site location: difference Fourier map
*R*[*F*^2^ > 2σ(*F*^2^)] = 0.043	Hydrogen site location: inferred from neighbouring sites
*wR*(*F*^2^) = 0.124	H-atom parameters constrained
*S* = 1.15	*w* = 1/[σ^2^(*F*_o_^2^) + (0.0646*P*)^2^ + 0.2701*P*] where *P* = (*F*_o_^2^ + 2*F*_c_^2^)/3
1203 reflections	(Δ/σ)_max_ < 0.001
100 parameters	Δρ_max_ = 0.27 e Å^−^^3^
0 restraints	Δρ_min_ = −0.23 e Å^−^^3^

### Special details

**Table:**

Experimental. The low-temperature data were collected with the Oxford Cyrosystems Cryostream low-temperature attachment.
Geometry. All e.s.d.'s (except the e.s.d. in the dihedral angle between two l.s. planes) are estimated using the full covariance matrix. The cell e.s.d.'s are taken into account individually in the estimation of e.s.d.'s in distances, angles and torsion angles; correlations between e.s.d.'s in cell parameters are only used when they are defined by crystal symmetry. An approximate (isotropic) treatment of cell e.s.d.'s is used for estimating e.s.d.'s involving l.s. planes.
Refinement. Refinement of *F*^2^ against ALL reflections. The weighted *R*-factor *wR* and goodness of fit *S* are based on *F*^2^, conventional *R*-factors *R* are based on *F*, with *F* set to zero for negative *F*^2^. The threshold expression of *F*^2^ > σ(*F*^2^) is used only for calculating *R*-factors(gt) *etc*. and is not relevant to the choice of reflections for refinement. *R*-factors based on *F*^2^ are statistically about twice as large as those based on *F*, and *R*- factors based on ALL data will be even larger.

### Fractional atomic coordinates and isotropic or equivalent isotropic displacement parameters (Å^2^)

**Table:**

	*x*	*y*	*z*	*U*_iso_*/*U*_eq_	
F1	0.24616 (17)	0.28505 (10)	0.59397 (12)	0.0409 (4)	
F2	0.29841 (19)	0.71120 (10)	0.45850 (14)	0.0434 (4)	
N1	0.4481 (2)	0.46153 (14)	0.79618 (16)	0.0297 (4)	
C1	0.5503 (3)	0.51558 (19)	0.9411 (2)	0.0334 (5)	
H1A	0.5590	0.6088	0.9317	0.040*	
H1B	0.6805	0.4809	0.9717	0.040*	
C2	0.3868 (2)	0.53796 (16)	0.6894 (2)	0.0277 (4)	
H2	0.4123	0.6256	0.7087	0.033*	
C3	0.2783 (2)	0.50109 (15)	0.53673 (18)	0.0244 (4)	
C4	0.2091 (2)	0.37948 (16)	0.49141 (19)	0.0272 (4)	
C5	0.1031 (2)	0.35046 (18)	0.3480 (2)	0.0311 (5)	
H5	0.0594	0.2664	0.3225	0.037*	
C6	0.0615 (3)	0.44634 (19)	0.2419 (2)	0.0332 (5)	
H6	−0.0123	0.4279	0.1426	0.040*	
C7	0.1257 (3)	0.56853 (19)	0.2780 (2)	0.0330 (5)	
H7	0.0974	0.6345	0.2052	0.040*	
C8	0.2317 (2)	0.59168 (16)	0.4228 (2)	0.0287 (4)	

### Atomic displacement parameters (Å^2^)

**Table:**

	*U*^11^	*U*^22^	*U*^33^	*U*^12^	*U*^13^	*U*^23^
F1	0.0569 (8)	0.0217 (6)	0.0346 (6)	−0.0067 (5)	−0.0046 (5)	0.0054 (4)
F2	0.0677 (8)	0.0190 (6)	0.0419 (7)	0.0003 (5)	0.0118 (6)	0.0036 (5)
N1	0.0339 (8)	0.0273 (8)	0.0247 (8)	0.0032 (6)	0.0022 (6)	−0.0038 (6)
C1	0.0331 (10)	0.0355 (10)	0.0280 (10)	−0.0021 (8)	0.0020 (8)	−0.0042 (8)
C2	0.0331 (9)	0.0210 (9)	0.0287 (9)	−0.0031 (7)	0.0077 (7)	−0.0021 (7)
C3	0.0259 (8)	0.0230 (9)	0.0248 (9)	0.0035 (7)	0.0075 (7)	−0.0012 (7)
C4	0.0306 (9)	0.0228 (9)	0.0271 (9)	0.0030 (7)	0.0059 (7)	0.0027 (7)
C5	0.0312 (9)	0.0292 (10)	0.0308 (10)	−0.0017 (7)	0.0044 (8)	−0.0059 (7)
C6	0.0300 (9)	0.0421 (11)	0.0251 (9)	0.0044 (8)	0.0032 (7)	−0.0014 (8)
C7	0.0370 (10)	0.0349 (10)	0.0278 (10)	0.0101 (8)	0.0098 (8)	0.0082 (8)
C8	0.0357 (10)	0.0198 (9)	0.0324 (10)	0.0042 (7)	0.0124 (8)	0.0007 (7)

### Geometric parameters (Å, °)

**Table:**

F1—C4	1.349 (2)	C3—C8	1.392 (2)
F2—C8	1.360 (2)	C3—C4	1.401 (3)
N1—C2	1.258 (2)	C4—C5	1.376 (3)
N1—C1	1.461 (2)	C5—C6	1.382 (3)
C1—C1^i^	1.502 (4)	C5—H5	0.950
C1—H1A	0.990	C6—C7	1.381 (3)
C1—H1B	0.990	C6—H6	0.950
C2—C3	1.471 (2)	C7—C8	1.374 (3)
C2—H2	0.950	C7—H7	0.950
			
C2—N1—C1	116.98 (16)	F1—C4—C3	118.52 (15)
N1—C1—C1^i^	110.12 (19)	C5—C4—C3	123.79 (16)
N1—C1—H1A	109.6	C4—C5—C6	118.53 (17)
C1^i^—C1—H1A	109.6	C4—C5—H5	120.7
N1—C1—H1B	109.6	C6—C5—H5	120.7
C1^i^—C1—H1B	109.6	C7—C6—C5	120.98 (17)
H1A—C1—H1B	108.1	C7—C6—H6	119.5
N1—C2—C3	124.56 (16)	C5—C6—H6	119.5
N1—C2—H2	117.7	C8—C7—C6	117.89 (18)
C3—C2—H2	117.7	C8—C7—H7	121.1
C8—C3—C4	114.00 (15)	C6—C7—H7	121.1
C8—C3—C2	120.03 (15)	F2—C8—C7	118.23 (17)
C4—C3—C2	125.95 (15)	F2—C8—C3	116.95 (16)
F1—C4—C5	117.69 (16)	C7—C8—C3	124.81 (17)
			
C2—N1—C1—C1^i^	119.1 (2)	C3—C4—C5—C6	−0.1 (3)
C1—N1—C2—C3	−178.90 (15)	C4—C5—C6—C7	0.4 (3)
N1—C2—C3—C8	−174.14 (17)	C5—C6—C7—C8	−0.1 (3)
N1—C2—C3—C4	7.6 (3)	C6—C7—C8—F2	178.75 (15)
C8—C3—C4—F1	−179.76 (15)	C6—C7—C8—C3	−0.6 (3)
C2—C3—C4—F1	−1.4 (3)	C4—C3—C8—F2	−178.50 (14)
C8—C3—C4—C5	−0.5 (3)	C2—C3—C8—F2	3.0 (2)
C2—C3—C4—C5	177.93 (16)	C4—C3—C8—C7	0.8 (3)
F1—C4—C5—C6	179.17 (15)	C2—C3—C8—C7	−177.67 (16)

Symmetry codes: (i) −*x*+1, −*y*+1, −*z*+2.

### Hydrogen-bond geometry (Å, °)

**Table:**

*D*—H···*A*	*D*—H	H···*A*	*D*···*A*	*D*—H···*A*
C5—H5···N1^ii^	0.95	2.53	3.471 (2)	171

Symmetry codes: (ii) *x*−1/2, −*y*+1/2, *z*−1/2.
